# The Fast Cooking and Enhanced Iron Bioavailability Properties of the Manteca Yellow Bean (*Phaseolus vulgaris* L.)

**DOI:** 10.3390/nu10111609

**Published:** 2018-11-01

**Authors:** Jason A. Wiesinger, Karen A. Cichy, Elad Tako, Raymond P. Glahn

**Affiliations:** 1 USDA-ARS, Robert W. Holley Center for Agriculture and Health, Cornell University, Ithaca, NY 14853, USA; Jason.Wiesinger@ars.usda.gov (J.A.W.); Elad.Tako@ars.usda.gov (E.T.); 2 USDA-ARS, Sugarbeet and Bean Research, Michigan State University, East Lansing, MI 48824, USA; Karen.Cichy@ars.usda.gov

**Keywords:** *Phaseolus vulgaris* L., yellow beans, Manteca, cooking time, iron, bioavailability, polyphenols, food security

## Abstract

The common dry bean (*Phaseolus vulgaris* L.) is a nutrient-dense pulse crop that is produced globally for direct human consumption and is an important source of protein and micronutrients for millions of people across Latin America, the Caribbean and Sub-Saharan Africa. Dry beans require large amounts of heat energy and time to cook, which can deter consumers worldwide from using beans. In regions where consumers rely on expensive fuelwood for food preparation, the yellow bean is often marketed as fast cooking. This study evaluated the cooking time and health benefits of five major market classes within the yellow bean seed type (Amarillo, Canary, Manteca, Mayocoba, Njano) over two field seasons. This study shows how the Manteca yellow bean possesses a fast cooking phenotype, which could serve as genetic resource for introducing fast cooking properties into a new generation of dry beans with cooking times <20 min when pre-soaked and <80 min unsoaked. Mineral analysis revealed fast cooking yellow beans have high iron retention (>80%) after boiling. An in vitro digestion/Caco-2 cell culture bioassay revealed a strong negative association between cooking time and iron bioavailability in yellow beans with r values = −0.76 when pre-soaked and −0.64 when unsoaked across the two field seasons. When either pre-soaked or left unsoaked, the highest iron bioavailability scores were measured in the fast cooking Manteca genotypes providing evidence that this yellow market class is worthy of germplasm enhancement through the added benefit of improved iron quality after cooking.

## 1. Introduction

Dry beans (*Phaseolus vulgaris* L.) are produced globally as a major pulse crop for direct human consumption. Biofortification efforts over the last decade focused primarily on developing new varieties of beans with increased iron concentrations adapted to thrive in Latin American and Sub-Sahara Africa [1]. The premise of iron biofortification is that more dietary iron will be available for absorption, thus alleviating iron deficiencies in regions where beans are a dietary staple [1,2]. Despite their capacity to be a rich source of iron, polyphenols in seed coats, high concentrations of phytate and thick cotyledon cell walls limit the bioavailability of iron from beans [3,4,5].

Cooking time is an additional factor that limits obtaining nutrients from beans, by simply discouraging bean consumption [6,7]. Long cooking times deter consumers from purchasing dry beans worldwide; especially in nations where energy needed for cooking is often expensive or scarce. Nearly three billion people in the world depend on traditional biomass, such as fuelwood or charcoal, as their main source of energy for cooking [8,9]. Regions where fuelwood is the primary source of energy are also the main areas with populations at risk for iron deficiencies, such as Sub-Sahara Africa, Central America or the Caribbean [10,11]. The problem is aggravated by widespread deforestation in these same nations, leading to dwindling stocks of fuelwood, and placing the burden of collecting cooking provisions principally on rural families [12,13]. The behavioral responses to fuelwood shortages in these communities are a significant impasse for using the bean as a biofortified crop to improve the nutritional well-being and food security of their inhabitants [14,15]. Research by Brouwer et al. demonstrated that as the scarcity of fuelwood increased, households of central Malawi would often postpone, or even omit energy-demanding beans from their meals and replace them with foods that required less fuelwood to cook [16,17].

There is great need for a fast cooking bean, which can positively impact consumers by reducing fuelwood needs, while simultaneously boosting the iron quality of meals [18,19,20]. An excellent opportunity to reduce the cooking time and improve the iron bioavailability of dry beans lies within the yellow bean seed type [21,22]. A vast number of shades and tones distinguish the yellow bean as a unique food crop, with “eye-catching” appeal in world marketplaces. While only a minor market class produced and sold in the United States, yellow beans are an important crop in Mexico, South America, and Sub-Saharan Africa, with a long history of domestication. Originating from the Peruano coast, over the millennium the yellow bean has diversified into a wide landscape of seed types, with many different shapes, sizes and market classes; facilitating their adaption into the traditional meals of communities worldwide [23]. At least a dozen different types of yellow beans are grown and sold throughout Latin America [23]. Yellow beans are also important in Africa, especially in Angola, Mozambique, Uganda, Tanzania and Zambia. Their popularity has been increasing in recent years and they often fetch the highest prices at the marketplace [24,25].

Notwithstanding their appeal to the modern day consumer, common bean breeding programs can also benefit from focusing on how yellow beans might distinguish themselves—nutritionally—from other bean market classes. The aim of this study was to examine the cooking quality, iron nutrition and iron absorption properties of the yellow bean seed type. A panel of yellow beans representing five market classes (Amarillo, Canary, Manteca, Mayocoba, Njano) that would be recognized by consumers in the marketplaces of Africa, the Americas or the Caribbean were evaluated for cooking time and iron density. An in vitro digestion/Caco-2 cell culture model was also used to measure iron bioavailability after cooking either pre-soaked or unsoaked beans from the panel.

## 2. Materials and Methods

### 2.1. The Yellow Bean Panel

**Figure 1:**
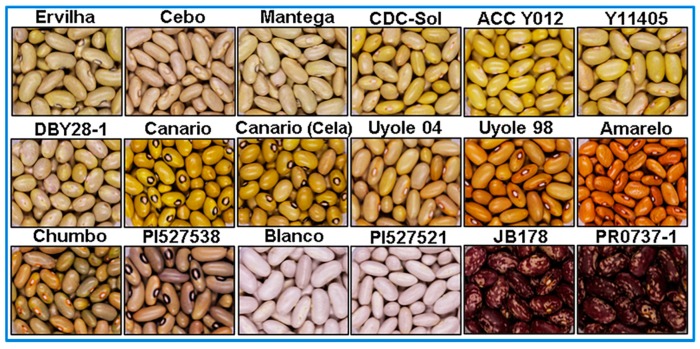
High-resolution photographs depicting the eighteen genotypes of the Yellow Bean Panel (YBP) arranged in order from lightest to darkest yellow seed coat color, followed by the white and red mottled controls. To compare differences in seed sizes, all photographs were taking to scale under standardized lighting conditions.

The Yellow Bean Panel (YBP) is a collection of 18 *P. vulgaris* genotypes selected to represent the five major market classes of the yellow bean seed type with geographic origins from East and South Africa, as well as North and South America. The market classes from lightest to darkest seed coat color include Manteca (pale yellow), Mayocoba (Peruano), Canary (bright yellow), Amarillo (yellow-orange) and Njano (yellow-green). A summary describing the collection sites, sources, cultivation status and gene pool of the YBP genotypes is presented in Table 1. Photographs of the YBP arranged from the lightest to darkest colored market classes are shown in Figure 1. The landraces Ervilha (Manteca) and Canario (Canary) were both collected from the Instituto de Investigação Agronómica located in the Huambo province of Angola. The landraces Cebo and Mantega Blanca (Manteca); Canario Cela (Canary); Chumbo (Njano); as well as the Middle American landrace, Amarelo (Amarillo) were all collected from the public marketplaces of Cuanza Sul province in Angola (Tim Porch, United States Department of Agriculture-Agricultural Research Service; USDA-ARS, Mayaguez, Puerto Rico; personal communication). The Njano, PI527538, was collected from Burundi. Genetic diversity analysis with single-nucleotide polymorphism (SNP) markers indicates this landrace is from the Andean gene pool and is likely a member of race Nueva Granada. The Njano and Soya Njano are preferred seed types grown in Eastern Africa [26] and are widely accepted for their agronomic performance, plant architecture and high yields (Susan Nchimbi-Msolla, Sokoine University of Agriculture; personal communication). Cultivars Uyole 98 and Uyole 04 were released in 1999 and 2004 by the Tanzanian National breeding program, renowned for their high yields, disease resistance, fast cooking properties and excellent ratings for palatability [27].

The North American Mayocoba seed types include CDC-Sol, which was released in 2013 and developed by the Crop Development Centre, University of Saskatchewan, Saskatoon; Saskatchewan [28]. This Canadian yellow is moderately resistance to Anthracnose (race 73), early maturing, and maintains its bright yellow color after storage [28]. AAC Y012 is an early maturing, high yielding yellow bean with partial field resistance to white mold, developed at the Agriculture and Agri-Food Canada (AAFC) Research and Development Centre located in Lethbridge, Alberta [29]. Y11405 is an advanced breeding line of the Michigan State University Dry Bean Breeding program. Y11405 is a North American adapted yellow bean with desirable end-use quality traits, such as a bright “highlighter” yellow seed coat and a consumer preference in seed size (James D. Kelly, Michigan State University; personal communication). DBY28-1 is a bean common mosaic virus (BCMV) and beet curly top virus (BCTV) resistance sister line to the early maturing yellow bean variety named “Patron”, which is a joint release of Oregon State University and the University of Idaho (James R. Myers, Oregon State University; personal communication). Four non-yellow *P. vulgaris* controls are also part of the YBP, which include a white bean landrace collected from Burundi (PI527521) and a white bean variety from Ecuador (Blanco Fanesquero). The other two controls include the red mottled JB178, a high yielding disease resistance variety released by the Dominican Republic in 1998 [30] and PR0737-1, a high yielding virus resistant red mottled line released jointly in 2013 by the University of Puerto Rico, USDA-ARS and the Haiti National Program [31]. The non-yellow controls were selected based upon their unique fast or slow cooking properties, which were measured during previous investigations [21,22,32].

### 2.2. Field Design and Storage Conditions

All YBP genotypes were planted in a Randomized-Complete-Block Design with 2 field replicates at the Michigan State University, Montcalm Research Farm near Entrican, MI in 2015 and 2016. Experimental units for each genotype consisted of two rows 4.75 m long with 0.5 m spacing between rows. Each experimental unit was separated by a broader row using the commercially available dark red kidney variety Red Hawk. The soil type is Eutric Glossoboralfs (coarse-loamy, mixed) and Alfic Fragiorthods (coarse-loamy, mixed, frigid). Rainfall was supplemented with overhead irrigation as needed. Daytime temperatures for field season 2015 averaged 76.4 °F and night temperatures averaged 54.7 °F. For field season 2016, daytime temperatures averaged 80.4 °F and night temperatures averaged 58.4 °F (www.usclimatedata.com). Weeds and pests were controlled throughout the season by hand, or with small amounts of herbicide if needed. Seed were harvested upon maturity by hand pulling the entire experimental unit and threshing with a Hege 140 plot harvester (Wintersteiger Inc., Salt Lake City, UT, USA). Immediately after harvest, bean seeds from each field replicate were hand sorted to eliminate any external material and any immature, wrinkled, discolored or damaged seeds. Sorted seed (moisture content 14–20%) was placed into dark storage under ambient conditions (20–22 °C, 50–60% relative humidity RH) at standard atmospheric pressure for six weeks. At this time, subsets of 100 randomly selected seeds from each field replicate were evaluated for cooking time, iron analysis and iron bioavailability. 

### 2.3. Moisture Equilibration, Cooking Time Determination and Sample Preparation

**Figure 2:**
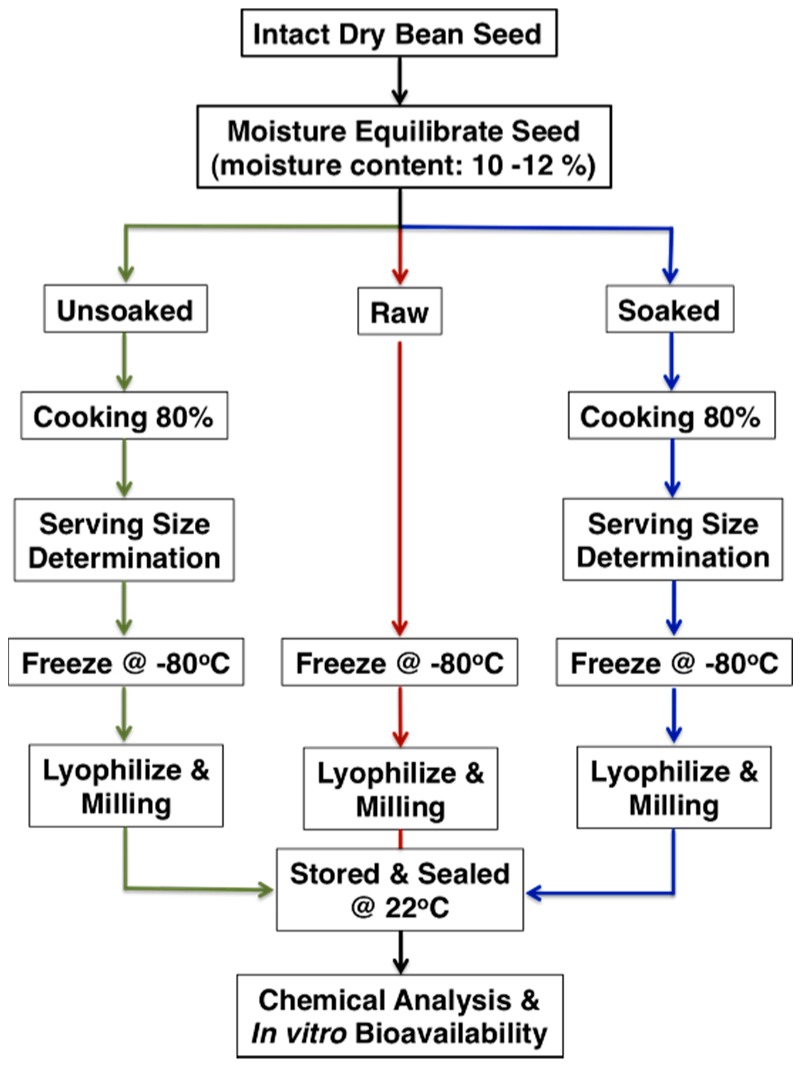
Flow diagram illustrating how cooking time is measured for bean seeds and how raw/cooked seed are processed for nutritional analysis and bioavailability assays.

To equilibrate moisture content after six weeks of storage, seeds were placed into paper envelopes and stored at room temperature until seed reached a moisture content range of 10–12% [33]. Prior to cooking, moisture-equilibrated bean seeds were either left unsoaked or soaked in distilled water (1:8 weight/weight) for 12 h at room temperature. Cooking time was determined using a Mattson pin drop cooking device fitted into a 4 L stainless steel beaker containing 1.8 L of boiling distilled water heated over a Waring SB30™ portable burner (Waring Commercial^®^, Torrington, CT, USA). Cooking time was standardized as the number of minutes required for 80% of 25 piercing tip rods (70 g, 2 mm diameter) to pass completely through each seed under a low-steady boil at 100 °C [34,35]. Once removed from boiling water, cooked seeds were cooled for 10 min at room temperature. For serving size determinations (defined as a half cup; 89 g wet weight) the number of cooked seed to fill a quarter cup (44.5 g, wet weight) was recorded, then doubled. Raw whole seed and their cooked whole seed counterparts were frozen at −80 °C before freeze-drying (VirTis Research Equip. Gardiner, NY, USA). To create a homogenous mixture of each genotype for chemical analysis, pre-weighed lyophilized raw seed and lyophilized cooked seed were ground into a fine powder with a Kinematica Polymix^®^ analytical mill (PX-MFC 90D, Bohemia, NY, USA) fitted with a 0.5 mm sieve followed by storage in sealed, opaque polypropylene plastic containers at 22 °C. A schematic illustrating the processing and cooking of the YBP is shown in Figure 2.

### 2.4. Iron Analysis

For iron analysis, 500 mg of lyophilized powder from raw and cooked seed was pre-digested in boro-silicate glass tubes with 3 mL of a concentrated ultra-pure nitric acid and perchloric acid mixture (60:40 *v*/*v*) for 16 h at room temperature. Samples were then placed in a digestion block (Martin Machine, Ivesdale, IL, USA) and heated incrementally over 4 h to a temperature of 120 °C with refluxing. After incubating at 120 °C for 2 h, 2 mL of concentrated ultra-pure nitric acid was subsequently added to each sample before raising the digestion block temperature to 145 °C for an additional 2 h. The temperature of the digestion block was then raised to 190 °C and maintained for at least ten minutes before samples were allowed to cool at room temperature. Digested samples were re-suspended in 20 mL of ultrapure water prior to analysis using ICP-AES (inductively coupled plasma-atomic emission spectroscopy; Thermo iCAP 6500 Series, Thermo Scientific, Cambridge, UK) with quality control standards (High Purity Standards, Charleston, SC, USA) following every 10 samples. Yttrium purchased from High Purity Standards (10M67-1) was used as an internal standard. To ensure batch-to-batch accuracy and to correct for matrix inference, all samples were digested and measured with 0.5 µg/mL of Yttrium (final concentration). The concentration of iron is expressed as the number of micrograms per gram of a lyophilized/milled powder that represents a homogeneous mixture of either 100 raw or 100 cooked seed for each YBP genotype.

### 2.5. Iron Content, Serving-Size, Dietary Reference Intake and Retention Values

To account for the intrinsic differences in seed sizes between the two field seasons and the extrinsic losses of seed mass during the cooking process, iron content was calculated for each genotype as the number of milligrams in 100 raw or 100 cooked seed. Iron contents are used to calculate serving size densities, by accounting for the number of cooked seed needed to fill a fixed serving volume [36]. Utilized by dietitians, nutrition researchers and practitioners in the United States, the USDA National Nutrient Database for Standard Reference (https://ndb.nal.usda.gov/ndb/) defines one serving of beans as a half of a cup, which equates to 89 g of cooked, drained and cooled whole seed (wet weight). Nutritional impact between the different genotypes of the YBP can be measured using the National Academy of Science’s Dietary Reference Intake (DRI) that is met with each serving of cooked seed [37]. Many initiatives sponsored by the U.S. Agency for International Development (USAID), U.S. State Department and World Health Organization (WHO) are focused on improving the health of vulnerable populations at risk to malnutrition, mainly women and children [19]. Therefore, the DRI values calculated in this study are based on the daily needs of an active adult female 19–50 years of age with a BMI ≤ 24 kg/m^2^ and an Estimated Energy Requirement (EER) of 2025 kcal/day [37]. Retention percentages were determined by comparing the total iron content between 100 raw and 100 cooked seeds. Iron content, serving size densities, DRI percentages and retention values are calculated according to the following formulas:

iron content = [iron concentration in lyophilized powder (mg/g)] × [average weight of lyophilized powder that represents 100 raw or cooked whole seeds (g/100 seed)](1)

(2) $$\mathrm{serving}\;\mathrm{size}\;=\;\frac{=\left[\mathrm{iron}\;\mathrm{content}\;\left(\mathrm{mg}/100\;\mathrm{seed}\right)\right]\;\times \;\left[\mathrm{number}\;\mathrm{of}\;\mathrm{seed}\;\mathrm{per}\;\mathrm{serving}\;\left(\mathrm{half}\;\mathrm{cup}\right)\right]}{\left[100\;\mathrm{seed}\right]}$$

(3) $$\%\;\mathrm{DRI}\;=\;\frac{\mathrm{milligrams}\;\mathrm{iron}\;\mathrm{per}\;\mathrm{serving}\;\left(\mathrm{mg}/\mathrm{half}\;\mathrm{cup}\right)}{\mathrm{milligrams}\;\mathrm{iron}\;\mathrm{required}\;\mathrm{per}\;\mathrm{day}\;\left(\mathrm{mg}/\mathrm{day}\right)}\;\times \;\left[100\%\right]$$

(4) $$\mathrm{retention}\;=\;\frac{\mathrm{cooked}\;\mathrm{iron}\;\mathrm{content}\;\left(g/100\;\mathrm{seed}\right)}{\mathrm{raw}\;\mathrm{iron}\;\mathrm{content}\;\left(g/100\;\mathrm{seed}\right)}\times \;\left[100\text{\%}\right]$$

### 2.6. Iron Bioavailability: In Vitro Digestion/Caco-2 Cell Bioassay

A 500 mg sample of lyophilized powder from cooked seed was subject to an in vitro digestion/Caco-2 cell culture model for the determination of iron bioavailability as described previously in Glahn et al., 1998 [38]. Iron uptake is measured as the increase in Caco-2 cell ferritin production (ng ferritin per milligram of total cell protein) following a simulated gastric and intestinal digestion, most recently described in Glahn et al., 2017 [39]. Iron bioavailability is expressed as a percentage score of Caco-2 cell ferritin formation that is relative to a control cooked/lyophilized/milled navy bean (variety Merlin). The navy bean control was run with each assay to index the ferritin/total cell protein ratios of the Caco-2 cells over the course of experimentation. Baseline ferritin values for the Caco-2 cells averaged 3.9 ± 1.6 ng/mg protein (mean ± Standard Deviation; SD) for 10 experiments spanning 3 months. Ferritin values for the Merlin navy bean control averaged 15 ± 4.7 ng/mg protein (mean ± SD). Ferritin values for the white bean control PI527521 averaged 14 ± 4.4, and the ferritin values for a blank digest with 66 µM FeCl_3_ averaged 64 ± 17 ng/mg protein (mean ± SD). The iron concentration of the cooked navy bean control over the course of experimentation averaged 76 ± 1.9 µg/g (mean ± SD).

### 2.7. Statistical Analysis

All statistical analyses were conducted using SAS 9.4 (SAS Institute Inc., Cary, NC, USA). The normality of the residuals for each parameter was evaluated using the Kolmogorov-Smirnov test. Measured parameters were found to have normal distribution, and were therefore acceptable for ANOVA without additional data transformation steps. Mean separations for genotypes were determined using the Proc MIXED procedure with the model including genotype (18 levels) and field season (2 levels) as fixed effects and field replicates (2 levels) as a random effect; followed by a Tukey *post hoc* test. Pearson correlation coefficients were calculated to determine the associations between measured variables and cooking time of the YBP. Differences with *p* values of ≤0.05 were considered statistically significant.

## 3. Results

### 3.1. Cooking Times and Cooking Classifications of the YBP

The cooking times of the eighteen YBP genotypes after soaking are listed in Table 2. The genotypes are ranked in Table 2 from fastest to slowest in one of three cooking classes: fast (<20 min), moderate (20–35 min) or slow (>35 min). Cooking time rank of all eighteen genotypes in YBP remained the same between the 2015 and 2016 field seasons (reported as combined means in Table 2). Year interactions (*p* = 0.257), as well as genotype × year interactions (*p* = 0.899) were not significant. A wide variation (*p* < 0.0001) in cooking times were measured among the yellow beans after soaking, ranging from 18–19 min for the three Manteca genotypes (Ervilha, Cebo, Mantega) to 69 min for the Middle American genotype Amarelo (Table 2). Significant variations (*p* < 0.0001) in cooking times were also measured between the yellow beans that were not soaked prior to cooking, ranging from 76–79 min for the three Manteca genotypes (Ervilha, Cebo, Mantega) to 126 min for Amarelo (Table 3). Unsoaked YBP genotypes listed in Table 3 are ranked from fastest to slowest in one of three cooking classes: fast (<80 min), moderate (80–110 min) or slow (>110 min). Year interactions and genotype × year interactions for cooking time were not significant among the unsoaked beans, and cooking time ranks were similar between the two field seasons. There was a strong relationship between the cooking times of the pre-soaked genotypes and the cooking times of the unsoaked genotypes in the YBP (*r* = 0.848, *p* < 0.0001). The cooking classifications of unsoaked genotypes, however, were not necessarily the same as pre-soaked genotypes (Table 2 and Table 3), and are presented as a separate grouping for each parameter measured in this study.

### 3.2. Iron Density of the YBP

Table 4 and Table 5 show the milligrams (mg) of iron provided in one serving of cooked beans from pre-soaked and unsoaked genotypes of the YBP organized from the fastest to slowest cooking. Iron DRI percentages for an adult female met with each serving of cooked beans are also shown in Table 4 and Table 5. The measurements used to determine the serving densities of iron in the soaked and unsoaked genotypes of the YBP, including the concentrations, contents and retention values of iron between the raw and cooked seed are presented in Tables S1–S5. Genotype, year interactions as well as genotype × year interactions for iron densities in the pre-soaked beans of the YBP were significant (*p* < 0.0001) after cooking. Serving densities ranged from 1.70 mg (9% of DRI) to 2.63 mg (15% of DRI) among the yellow beans across the 2015 and 2016 field seasons (Table 4). High serving densities of iron (14–16% of DRI) were measured in both the red mottled varieties JB178 and PR0737-1 in 2015 and in 2016. The yellow breeding line Y11405 had the highest serving density of iron among the yellow beans (14–15% of DRI) for both field seasons (Table 4). There was no relationship between the cooking times and the iron densities of pre-soaked genotypes in the YBP for either the 2015 (*r* = 0.221, *p* = 0.299) and 2016 (*r* = −0.134, *p* = 0.533) field seasons.

The milligrams (mg) of iron provided in one serving of cooked beans from unsoaked genotypes of the YBP are shown in Table 5. Genotype, year interactions and genotype × year interactions for iron densities among the unsoaked bean samples were significant (*p* < 0.0001). Table 5 shows the serving densities of iron ranged from 1.39 mg (8% of DRI) to 2.50 mg (14% of DRI) among the yellow bean genotypes in both the 2015 and 2016 field season. The highest serving densities of the iron (2.35–2.50 mg; 13–14% of DRI) were measured in the red mottled variety JB178 and the yellow breeding line Y11405 (Table 5). There was no relationship between the cooking times and the iron densities of the unsoaked genotypes for field seasons 2015 (*r* = 0.127, *p* = 0.556) and 2016 (*r* = 0.393, *p* = 0.058).

### 3.3. Iron Retention Values of the YBP

The content and retention values for iron in 100 raw and 100 cooked seed of the YBP are presented in Tables S4 and S5. Genotype, year interactions as well as genotype × year interactions for iron retention after cooking the pre-soaked and unsoaked genotypes of the YBP were significant (*p* < 0.0001). After soaking and cooking the YBP, iron retention values ranged from 77–91% across the 2015 and 2016 field seasons (Table S4). High retention values for iron (83–91%) were measured in the three fast cooking Manteca yellow beans (Table S4), and there was a significant relationship between the cooking times of the YBP and retention of iron in both the 2015 (*r* = −0.659, *p* = 0.0001) and 2016 (*r* = −0.572, *p* = 0.003) field seasons.

Iron retention values in the unsoaked and cooked YBP genotypes ranged from 71–85% across the 2015 and 2016 field seasons (Table S5). Higher retention values for iron (80–84%) were measured in the fast cooking Manteca yellows when compared to the slow cooking yellow beans (Table S5). There was a strong relationship between the retention of iron and the cooking times of the eighteen unsoaked YBP genotypes in 2015 (*r* = −0.789, *p* < 0.0001) and 2016 (*r* = −0.729, *p* < 0.0001).

### 3.4. Iron Bioavailability of the YBP

**Figure 3:**
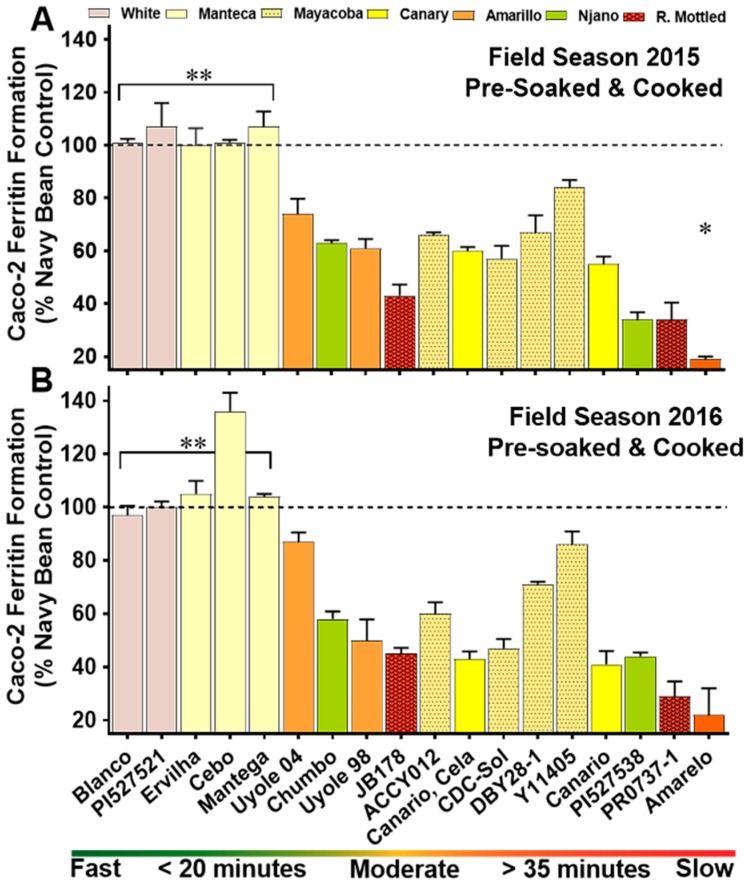
Iron bioavailability scores of pre-soaked and cooked whole seed genotypes in the YBP for field season 2015 (**A**) and field season 2016 (**B**). Values are means (±Standard Deviation) of triplicate measurements from two field replicates per genotype (*n* = 6). Genotypes are categorized on the x-axis by cooking class, ranked from the fastest cooking genotype to slowest cooking entry. * Significantly lower (*p* ≤ 0.05) iron bioavailability score when compared to the other YBP entries. ** Significantly higher (*p* ≤ 0.05) iron bioavailability scores compared to the other YBP genotypes.

The results illustrated in Figure 3 and listed with mean separations in Table S6 show significant variations (*p* < 0.0001) in the percentage scores of iron bioavailability after cooking the pre-soaked genotypes of the YBP. Year interactions as well as genotype × year interactions for iron bioavailability in pre-soaked/cooked beans of the YBP were significant (*p* < 0.0001). In 2015, iron bioavailability scores as a percent of the navy bean control ranged from as low as 19% in the slow cooking Amarelo to a high of 107% in the fast cooking Manteca landrace, Ervilha (Figure 3A). Similar variations in iron bioavailability among the YBP genotypes were also measured in 2016, ranging from 22% in Amarelo to 136% in Cebo, the fast cooking Manteca (Figure 3B). When compared to the other moderate and slow cooking genotypes in the YBP, the fast cooking white bean controls and Manteca genotypes had significantly higher iron bioavailability scores (Figure 3). Iron bioavailability was strongly correlated with the cooking times of pre-soaked YBP genotypes in 2015 (*r* = −0.814, *p* < 0.0001) and 2016 (*r* = −0.737, *p* < 0.0001). Iron bioavailability scores were low in red mottled varieties JB178 and PR0737-1, ranging from only 29–45% across the 2015 and 2016 field seasons (Figure 3).

**Figure 4:**
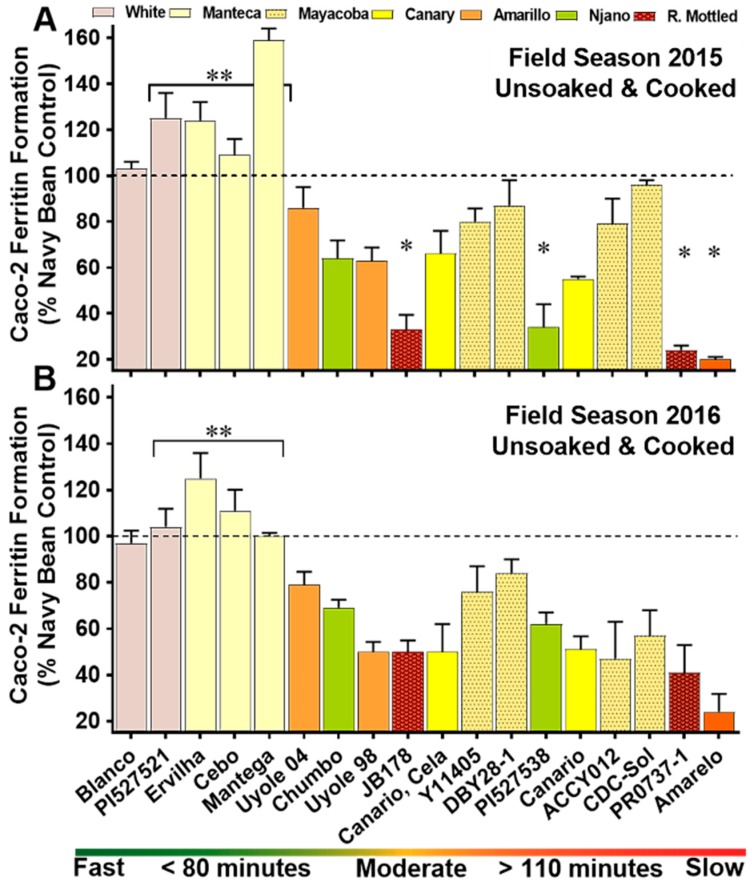
Iron bioavailability scores of unsoaked and cooked whole seed genotypes in the YBP for field season 2015 (**A**) and field season 2016 (**B**). Values are means (±Standard Deviation) of triplicate measurements from two field replicates per genotype (*n* = 6). Genotypes are categorized on the x-axis by cooking class, ranked from the fastest cooking genotype to slowest cooking entry. * Significantly lower (*p* ≤ 0.05) iron bioavailability score when compared to the other YBP entries. ** Significantly higher (*p* ≤ 0.05) iron bioavailability scores compared to the other YBP genotypes.

Significant variations (*p* < 0.0001) in iron bioavailability were also measured after cooking the unsoaked genotypes of the YBP (Figure 4; Table S7). Year interactions and genotype × year interactions were significant (*p* < 0.0001) with iron bioavailability scores ranging from a low of 20% in the slow cooking Amarelo to as high as 159% in the fast cooking Mantega Blanca across the 2015 and 2016 field seasons (Figure 4). For the unsoaked and cooked genotypes in the YBP, the highest iron bioavailability scores were measured in the fast cooking three Manteca genotypes, while the lowest scores for iron bioavailability were measured in the slow cooking red mottled PR0737-1 and the slow cooking yellow Amarelo (Figure 4). There was a strong relationship between the cooking times and iron bioavailability of unsoaked YBP genotypes in 2015 (*r* = −0.705, *p* < 0.0001) and 2016 (*r* = −0.604, *p* < 0.001).

## 4. Discussion

### 4.1. The YBP Is a Model to Explore the Health Benefits Yellow Beans

The YBP includes a diverse set of landraces, varieties and breeding lines within five yellow bean market classes. The model takes into consideration how different cultures around the world traditionally prepare beans for cooking: by either soaking or not soaking prior to boiling [6,40]. The two white beans from Burundi and Ecuador were selected as non-yellow controls because of their fast cooking properties [22]. The two red mottled beans from the Caribbean were selected as non-yellow controls because of their ability to acquire high concentrations of iron at the Montcalm Research Farm in Michigan. They also have contrasting fast (JB178) and slow (PR0737-1) cooking properties [21]. White and red mottled beans are on opposite ends of the iron bioavailability spectrum for dry beans [41,42], creating the ideal framework for evaluating the iron quality of the different yellow beans in the YBP.

Information on dry bean nutrition is most often reported on raw seed, which is first milled into a powder, then dried to remove moisture [43,44]. This study is unique because the nutritional evaluation was conducted after cooking, allowing for the genotypic differences in nutrient retention to be expressed in the model. Raw seed analysis of the dry bean does not take into consideration the genetic variability in (1) the loss of total seed mass during cooking process, (2) the retention of nutrients after cooking and (3) the size of hydrated seed in a fixed volume for the calculation serving size density [21,45,46,47]. Minerals in dry beans are particularly sensitive to long cooking times [21,35]. Even under the standardized conditions of this study, the losses of iron in the yellow beans were not trivial after cooking. Retention values below 75% for iron were measured in the slowest cooking genotypes of the YBP, especially when the cooking times are extended in the unsoaked seed (Tables S4 and S5).

For breeding programs, advancing new traits into the next generation of food crops depends on access to a large collection of diverse germplasm [48]. Although beneficial alleles can be introduced between different the market classes of *P. vulgaris* (e.g., white bean crossed to a red mottled), common bean breeding programs focus on crosses within a market class because of the challenge to maintain the appropriate combination of genes for seed size, shape and color [44,49]. The YBP model shows there is wide diversity in consumer friendly traits to explore within the yellow bean market classes. To increase the consumption and health promoting properties of beans worldwide, consumer targeted traits, such as fast cooking times and boosted nutritional value are now being considered in addition to the new cultivar’s strong agronomic performance [19,20].

### 4.2. The Manteca Yellow Bean: A Genetic Resource for the New Generation of Fast Cooking Andean Beans

The Manteca is a pale lemon colored seed native to Chile, where traditional knowledge describes the Manteca as an “easy-to-digest” bean with low flatulence [50,51,52]. The three Manteca landraces collected from Angola had fast cooking times when either soaked or left unsoaked for both the 2015 and 2016 field season. Two previous studies have also identified the Manteca as a fast cooking yellow bean when grown at the Montcalm Research Farm, cooking in less than 25 min under a set of standardized storage and soaking conditions over the course of the 2012–2013 field seasons [21,22]. With a set of nearly 5000 polymorphic SNPs, Nei genetic distance [53] on 206 genotypes of *P. vulgaris* revealed a phylogenetic relationship between the Manteca landraces and other fast cooking beans, including the white bean control PI527521 from Burundi and a fast cooking cranberry bean (G23086) from Malawi [22]. The genetic relatedness of these genotypes suggests a common genetic control for the fast cooking phenotype. Their origins are from regions in Africa where fuelwood is the major source of energy for cooking, which could explain why farmers valued and maintained the fast cooking trait within these landraces [22]. What impact the environment might play on the genetic expression of the fast cooking phenotype is still under investigation.

Specific genetic mechanisms that control the cooking time of *P. vulgaris* have yet to be identified. How different morphological features of a bean seed influence cooking time could be the clue to what underlying genetic mechanisms might be involved. The surface area and shape of the seed, as well as the thickness and chemical composition of the seed coat can affect the water uptake and the cooking time of dry beans [54,55,56]. The expression of flavanol glycosides, anthocyanins and condensed tannins in seed coats not only leverages color, but also contribute to the hydration and cooking properties of dry beans [57]. Previous research shows there is a strong positive correlation (r = 0.77) between cooking time and seed tannin content in dry beans [58]. More recent research demonstrates after soaking and boiling, fast cooking beans have higher soluble dietary fiber concentrations when compared to their slow cooking counterparts from yellow, cranberry, red mottled and light red kidney market classes [32]. These findings suggest the physical and chemical composition of the fast cooking dry bean may be unique, and might have a common genetic architecture.

### 4.3. Iron Nutrition Benefits of the Fast Cooking Manteca Yellow Bean

Environmental factors, such as precipitation, drought stress and soil characteristics affect the mineral concentrations of dry beans [44,59]. The iron nutrition of the YBP was diverse, and there was a significant year and genotype × year interaction. There was no relationship between the cooking times of the genotypes in the YBP and the intrinsic concentrations of iron in their raw seed. The amount of iron retained after cooking, however, was strongly associated with cooking time in the YBP. Although the Manteca landraces did not have high iron concentrations in their raw seed when compared to other yellow and red mottled genotypes in the YBP, their fast cooking properties contribute to an improved nutritional value through the benefit of high iron retention during the cooking process (Tables S4 and S5).

There was a large genotype and genotype × year interaction for iron bioavailability in the YBP, with many of the yellows performing just as poorly as the low iron bioavailable red mottled controls. The iron bioavailability of YBP was independent of iron concentrations in raw and cooked seed. A strong relationship was detected between cooking time and iron bioavailability in the YBP. The light colored and faster cooking Uyole 04 outperformed the darker orange Amarillo’s (Uyole 98, Amarelo); suggesting that a darker seed coat color may be contributing to lower iron bioavailability [4,41,42]. The same observation was previously demonstrated in a separate cooking model for dry beans that examined fast, moderate and slow cooking genotypes from four different market classes of economic importance in Africa, the Americas and the Caribbean [21]. The evidence is building that breeding for fast cooking times may have the added benefit of improving the iron absorption properties in dry beans. Whether pre-soaked or left unsoaked, the fast cooking Mantecas distinguish themselves from the other yellow seed types in the YBP with the highest iron bioavailability scores measured in both the 2015 and 2016 field seasons. Not soaking the Manteca yellow beans prior to boiling did not negatively impact their iron bioavailability scores. This is an important feature of the Manteca to note, because many cultures in Africa, Latin America and the Caribbean do not soak their beans before cooking because it alters the flavor [6,40].

### 4.4. Profile of the Manteca

New questions arise in understanding how the alleged digestibility of the Mantecas might be related to their high iron bioavailability. The antidotal claim of the “easy-to-digest” Manteca bean was first investigated by British agriculture scientist Colin Leaky (1933–2018), who noticed the more expensive Manteca in the markets of Chile in the late 1970′s, lauded by traders as “beans for the rich man’s table” [51]. A decade earlier, Dr. Leaky was challenged by nutritionists in Uganda to help improve the nutrient quality of meals by breeding a more digestible bean for babies to tolerant as a first food [60]. Leakey was successful in releasing Prim (named after the saying “Prim and Proper”) a modern Manteca variety with low-flatulence and excellent flavor [60,61]. Indeed, there is evidence to support the Manteca yellow bean may have a unique nutritional profile compared to other beans: with less dietary fiber, less indigestible protein and starch, but with similar concentrations of oligosaccharides [32,52,61,62,63]. Manteca beans are also free of proanthocyanins and condensed tannins—classes of compounds shown to reduce protein digestibility and iron absorption [3,64,65].

Secondary metabolites in beans, such as phytate and certain polyphenolic compounds, can inhibit the absorption of iron [3,4,66]. Yellow beans with the Prim heritage are believed to carry a recessive allele that shifts the polyphenolic pathway in seed coats away from tannin and proanthocyanin synthesis towards the accumulation of kaemperfol derived flavonoids, primarily keampferol-3-glucoside [50,64]. Iron uptake assays with Caco-2 cells have recently demonstrated that kaemperfol and kaemperfol-3-glucoside are actually promoters of iron absorption. In contrast, polyphenols expressed in the seed coats of red or black beans, such as quercetin or myricetin act as strong inhibitors to iron absorption [4,66]. As an example to support these findings, the Canary colored yellow beans in the YBP (Canario, Canario, Cela) expresses a dominant form of this allele in their seed coats, opening the biosynthetic pathway for the production of iron inhibitory polyphenols, such as procyanidins and quercetin 3-glucoside [67,68]. In both the 2015 and 2016 field seasons, the two Canary genotypes (Canario, Canario, Cela) had higher iron concentrations in their cooked seed (Tables S2 and S3), but had significantly lower iron bioavailability scores when compared to the Manteca landraces Ervilha, Cebo and Mantega (Figure 3 and Figure 4). The secret of improved iron bioavailability in the Manteca may be revealed by the unique polyphenolic pattern expressed in their seed coats. Detailed studies examining the polyphenolic profile and how they might be related to the different iron bioavailability properties of the yellow, white and red mottled genotypes in the YBP are currently being conducted.

## 5. Conclusions

A sustainable public breeding effort is under way to increase the global production and health benefits of the common dry bean. The purpose of this study was to explore five of the major yellow bean market classes for promising phenotypes that can be added to the next generation of dry beans. The Manteca yellow bean is certainly a prize of the Andean gene pool. This study provides evidence that the Manteca is a nutritionally viable target for germplasm enhancement through the added benefit of fast cooking times and improved iron bioavailability. The hope is the yellow bean can be used to encourage more bean consumption by appealing to the consumers through traits not given a priority in other bean market classes, such as fast cooking time for convenience or improved iron quality for nutrition. Manteca beans formulated into bean-based diets for a long-term in vivo feeding trial is the next step in evaluating the iron benefits of this market class beyond the current in vitro assessment presented in this study.

## Figures and Tables

**Table 1:** Description, Collection Sites, Source, Cultivation Status and Center of Domestication (COD) of the Eighteen Genotypes that Characterize the Yellow Bean Panel (YBP) ^1^.

Seed Type	Genotype	Collection Site	Source	Cultivation	COD
Manteca	Ervilha	IIA	Huambo, Angola	Landrace *	Andean
Manteca	Cebo	marketplace	Cela, Angola	Landrace *	Andean
Manteca	Mantega Blanca	marketplace	Kibala, Angola	Landrace *	Andean
Mayocoba	CDC-Sol	Canada	Unv. of Saskatchewan	Variety	Andean
Mayocoba	ACC Y012	Canada	Alberta	Variety	Andean
Mayocoba	Y11405	United States	Michigan State Unv.	Breeding Line	Andean
Mayocoba	DBY28-1	United States	Oregon State Unv.	Breeding Line	Andean
Canary	Canario	IIA	Huambo, Angola	Landrace *	Andean
Canary	Canario, Cela	marketplace	Cela, Angola	Landrace *	Andean
Amarillo (*lt*.)	Uyole 04	Tanzania	Tanzania Breeding	Variety	Andean
Amarillo (*dk.*)	Uyole 98	Tanzania	Tanzania Breeding	Variety	Andean
Amarillo (*dk*.)	Amarelo	marketplace	Cela, Angola	Landrace *	MA
Njano	Chumbo	marketplace	Cela, Angola	Landrace *	Andean
Njano	PI527538	Burundi	US GRIN	Landrace	Andean
White	PI527521	Burundi	US GRIN	Landrace	Andean
White	Blanco Fanesquero	Ecuador	INIAP	Variety	Andean
Red Mottled	JB178	Dominican Rep.	CIAS	Variety	Andean
Red Mottled	PR0737-1	Puerto Rico	Unv. of Puerto Rico	Variety	Andean

^1^ The YBP consists of medium to large Andeans ranging from 40–65 g/100 seed, and a small Middle American (MA) averaging 30 g/100 seed. Genotypes are arranged from the lightest to the darkest yellow seed types, followed by the white and red mottled controls. * Not verified as landraces; accessions collected from provinces located in Angola, Africa. IIA, Instituto de Investigação Agronómica; US GRIN, U.S. Germplasm Resources Information Network; INIAP, Instituto Nacional de Investigaciones Agropecuarias; CIAS, Centro de Investigación Agricolas del Suroeste. (*lt*.) light yellow; (*dk*.) dark yellow.

**Table 2:** Cooking Times of Pre-Soaked Genotypes in the Yellow Bean Panel ^1^.

Genotype (*Seed Type*)	Cooking Time (min) ^2^	Cooking Class
Blanco (*white*)	16 ^k^	fast
PI527521 (*white*)	18 ^k^	fast
Ervilha (*Manteca*)	18 ^j,k^	fast
Cebo (*Manteca*)	19 ^j,k^	fast
Mantega (*Manteca*)	19 ^j,k^	fast
Uyole 04 (*lt. Amarillo*)	22 ^i,j^	moderate
Chumbo (*Njano*)	24 ^h,i^	moderate
Uyole 98 (*dk. Amarillo*)	26 ^f,g,h^	moderate
JB178 (*Red Mottled*)	26 ^g,h^	moderate
ACC Y012 (*Mayocoba*)	28 ^e,f,g^	moderate
Canario, Cela (*Canary*)	29 ^e,f,g^	moderate
CDC-Sol (*Mayocoba*)	30 ^d,e,f^	moderate
DBY28-1 (*Mayocoba*)	31 ^d,e^	moderate
Y11405 (*Mayocoba*)	33 ^d^	moderate
Canario (*Canary*)	38 ^c^	slow
PI527538 (*Njano*)	39 ^c^	slow
PR0737-1 (*Red Mottled*)	59 ^b^	slow
Amarelo (*dk. Amarillo*)	69 ^a^	slow

^1^ Values are combined means of duplicate measurements from two field replicates per genotype (*n* = 4) for field seasons 2015 and 2016. Means sharing the same letter are not significantly different at *p* ≤ 0.05. ^2^ Raw seed were soaked in distilled water for 12 h prior to determining the number of minutes to reach 80% cooking time with an automated Mattson pin-drop device, then categorized top to bottom from the fastest to slowest cooking entry. (*lt*.) light yellow; (*dk*.) dark yellow.

**Table 3:** Cooking Times of Unsoaked Genotypes in the Yellow Bean Panel ^1^.

Genotype (*Seed Type*)	Cooking Time (min) ^2^	Cooking Class
Blanco (*white*)	76 ^k,l^	fast
PI527521 (*white*)	76 ^j,k,l^	fast
Ervilha (*Manteca*)	76 ^l^	fast
Cebo (*Manteca*)	76 ^l^	fast
Mantega (*Manteca*)	79 ^i,j,k^	fast
Uyole 04 (*lt. Amarillo*)	82 ^h,i,j^	moderate
Chumbo (*Njano*)	83 ^h^	moderate
Uyole 98 (*dk. Amarillo*)	83 ^h,i^	moderate
JB178 (*Red Mottled*)	95 ^g^	moderate
Canario, Cela (*Canary*)	101 ^f^	moderate
Y11405 (*Mayocoba*)	101 ^f^	moderate
DBY28-1 (*Mayocoba*)	108 ^d,e^	moderate
PI527538 (*Njano*)	108 ^e^	moderate
Canario (*Canary*)	112 ^c,d^	slow
ACC Y012 (*Mayocoba*)	113 ^b,c^	slow
CDC-Sol (*Mayocoba*)	116 ^b^	slow
PR0737-1 (*Red Mottled*)	124 ^a^	slow
Amarelo (*dk. Amarillo*)	126 ^a^	slow

^1^ Values are combined means of duplicate measurements from two field replicates per genotype (*n* = = 4) for field seasons 2015 and 2016. Means sharing the same letter are not significantly different at *p* ≤ 0.05. ^2^ Raw seed were left unsoaked prior to determining the number of minutes to reach 80% cooking time with an automated Mattson pin-drop device, then categorized top to bottom from the fastest to slowest cooking entry. (*lt*.) light yellow; (*dk*.) dark yellow.

**Table 4:** Cooked Seed Iron Density of Pre-Soaked Genotypes in the Yellow Bean Panel Organized by Cooking Class ^1^.

		One Serving Size (Half Cup)			
		2015		2016	
Genotype (*Seed Type*)	Cooking Class	Iron (mg) ^2^	% DRI ^3^	Iron (mg)	% DRI
Blanco (*white*)	fast	1.95 ^d,e,f^	11	2.28 ^b,c,d,e^	13
PI527521 (*white*)	fast	2.13 ^c,d^	12	2.32 ^b,c,d^	13
Ervilha (*Manteca*)	fast	2.02 ^d,e^	11	2.30 ^b,c,d,e^	13
Cebo (*Manteca*)	fast	1.75 ^f,g^	10	2.02 ^g,h^	11
Mantega (*Manteca*)	fast	2.06 ^c,d^	11	2.29 ^b,c,d,e^	13
Uyole 04 (*lt. Amarillo*)	moderate	1.84 ^e,f,g^	10	2.16 ^d,e,f,g^	12
Chumbo (*Njano*)	moderate	1.98 ^d,e^	11	2.25 ^c,d,e,f^	12
Uyole 98 (*dk. Amarillo*)	moderate	1.85 ^e,f,g^	10	2.06 ^f,h,g^	11
JB178 (*Red Mottled*)	moderate	2.71 ^a^	15	2.89 ^a^	16
ACC Y012 (*Mayocoba*)	moderate	1.84 ^e,f,g^	10	2.10 ^e,f,g^	12
Canario, Cela (*Canary*)	moderate	2.24 ^c^	12	2.30 ^b,c,d,e^	13
CDC-Sol (*Mayocoba*)	moderate	1.82 ^e,f,g^	10	1.95 ^g,h^	11
DBY28-1 (*Mayocoba*)	moderate	1.73 ^g^	10	2.02 ^g,h^	11
Y11405 (*Mayocoba*)	moderate	2.63 ^a,b^	15	2.49 ^b^	14
Canario (*Canary*)	slow	1.98 ^d,e^	11	2.14 ^d,e,f,g^	12
PI527538 (*Njano*)	slow	1.71 ^g^	10	1.87 ^h^	10
PR0737-1 (*Red Mottled*)	slow	2.49 ^b^	14	2.45 ^b,c^	14
Amarelo (*dk. Amarillo*)	slow	1.70 ^g^	9	2.02 ^g,h^	11

^1^ Values are means of duplicate measurements from two field replicates per genotype (*n* = 4), measured for field seasons 2015 and 2016. Means sharing the same letter in each column are not significantly different at *p* ≤ 0.05. ^2^ Average grams of iron measured in a half cup (89 g, wet weight) of cooked drained whole seed that were first soaked in distilled water for 12 h prior to determining the number of minutes to reach 80% cooking time. ^3^ Percent of daily reference intake met for iron (18 mg) of an adult female (19–50 years) measured in each serving of cooked whole seed. (*lt*.) light yellow; (*dk*.) dark yellow.

**Table 5:** Cooked Seed Iron Density of Unsoaked Genotypes in the Yellow Bean Panel Organized by Cooking Class ^1^.

		One Serving Size (Half Cup)			
		2015		2016	
Genotype (*Seed Type*)	Cooking Class	Iron (mg) ^2^	% DRI ^3^	Iron (mg)	% DRI
Blanco (*white*)	fast	2.07 ^c,d^	11	2.24 ^b,c,d^	12
PI527521 (*white*)	fast	1.98 ^d,e^	11	2.17 ^b,c,d,e,f^	12
Ervilha (*Manteca*)	fast	2.19 ^b,c^	12	2.20 ^b,c,d,e^	12
Cebo (*Manteca*)	fast	1.62 ^i,j^	9	2.00 ^e,f,g,h^	11
Mantega (*Manteca*)	fast	1.85 ^e,f^	10	2.01 ^e,f,g,h^	11
Uyole 04 (*lt. Amarillo*)	moderate	1.68 ^f,g,h,i,j^	9	2.12 ^d,e,f,g^	12
Chumbo (*Njano*)	moderate	1.83 ^e,f,g^	10	1.95 ^g,h,i^	11
Uyole 98 (*dk. Amarillo*)	moderate	1.79 ^f,g,h^	10	1.98 ^f,g,h,i^	11
JB178 (*Red Mottled*)	moderate	2.43 ^a^	13	2.49 ^a^	14
Canario, Cela (*Canary*)	moderate	2.25 ^b^	12	2.14 ^d,e,f,g^	12
Y11405 (*Mayocoba*)	moderate	2.50 ^a^	14	2.35 ^a,b^	13
DBY28-1 (*Mayocoba*)	moderate	1.65 ^h,i,j^	9	1.90 ^h,i^	11
PI527538 (*Njano*)	moderate	1.60 ^j^	9	1.79 ^i^	10
Canario (*Canary*)	slow	2.01 ^d^	11	2.04 ^e,f,g,h^	11
ACC Y012 (*Mayocoba*)	slow	1.68 ^g,h,i,j^	9	1.89 ^h,i^	10
CDC-Sol (*Mayocoba*)	slow	1.77 ^f,g,h,i^	10	1.83 ^h,i^	10
PR0737-1 (*Red Mottled*)	slow	2.11 ^b,c,d^	12	2.28 ^a,b,c^	13
Amarelo (*dk. Amarillo*)	slow	1.56 ^j^	9	1.39 ^j^	8

^1^ Values are means of duplicate measurements from two field replicates per genotype (*n* = 4), measured for field seasons 2015 and 2016. Means sharing the same letter in each column are not significantly different at *p* ≤ 0.05. ^2^ Average grams of iron measured in a half cup (89 g, wet weight) of cooked drained whole seed that were left unsoaked prior to determining the number of minutes to reach 80% cooking time. ^3^ Percent of daily reference intake met for iron (18 mg) of an adult female (19–50 years) measured in each serving of cooked whole seed. (*lt*.) light yellow; (*dk*.) dark yellow.

## Supplementary Materials

The following are available online at http://www.mdpi.com/2072-6643/10/11/1609/s1, Table S1, raw seed iron concentrations. Table S2, Iron Concentrations of Pre-Soaked and Cooked Genotypes in the Yellow Bean Panel Organized by Cooking Class. Table S3, Iron Concentrations of Unsoaked and Cooked Genotypes in the Yellow Bean Panel Organized by Cooking Class. Table S4, Iron Contents and Retention Values of Pre-Soaked and Cooked Genotypes in the Yellow Bean Panel Organized by Cooking Class. Table S5, Iron Contents and Retention Values of Unsoaked and Cooked Genotypes in the Yellow Bean Panel Organized by Cooking Class. Table S6, Iron Bioavailability Scores of Pre-Soaked and Cooked Genotypes in the Yellow Bean Panel Organized by Cooking Class. Table S7, Iron Bioavailability Scores of Unsoaked and Cooked Genotypes in the Yellow Bean Panel Organized by Cooking Class.
