# Supplementary material for: The Fast Cooking and Enhanced Iron Bioavailability Properties of the Manteca Yellow Bean (Phaseolus vulgaris L.)
Source: Nutrients. 2018 Nov 1;10(11):1609. doi: 10.3390/nu10111609 (PMC6266362; doi:10.3390/nu10111609)
Supplement: Supplementary file 1 [file nutrients-10-01609-s001.zip › nutrients-369272-SI.pdf]

Nutrients 2018, 10, 1609; doi:10.3390/nu10111609 www.mdpi.com/journal/nutrients

Table S1. Raw Seed Iron Concentrations of the Eighteen Genotypes in the Yellow Bean Panel Organized from Lightest to Darkest Seed Type.[1]

	**Raw Seed Iron (μg/g)[2]**	
**Genotype (*Seed Type*)**	2015	2016
Blanco (*white*)	59[gh]	71[def]
PI527521 (*white*)	66[cd]	73[cde]
Ervilha (*Manteca*)	55[hi]	65[hij]
Cebo (*Manteca*)	60[fg]	69[efg]
Mantega (*Manteca*)	60[fg]	62[j]
CDC-Sol (*Mayocoba*)	58[gh]	62[j]
ACC Y012 (*Mayocoba*)	64[def]	62[j]
Y11405 (*Mayocoba*)	80[a]	79[ab]
DBY28-1 (*Mayocoba*)	54[i]	63[ij]
Canario (*Canary*)	68[bc]	76[bc]
Canario, Cela (*Canary*)	70[b]	69[fgh]
Uyole 04 (*lt. Amarillo*)	53[i]	64[ij]
Uyole 98 (*dk. Amarillo*)	61[fg]	66[ghi]
Amarelo (*dk. Amarillo*)	65[cde]	73[cd]
Chumbo (*Njano*)	62[f]	65[ij]
PI527538 (*Njano*)	56[hi]	66[ghi]
JB178 (*Red Mottled*)	79[a]	81[a]
PR0737-1 (*Red Mottled*)	79[a]	80[a]

[1]Values are means of duplicate measurements from two field replicates per genotype (n = 4), measured for field seasons 2015 and 2016. Means sharing the same letter in each column are not significantly different at $P \leq 0.05$. [2]Total iron concentrations expressed as micrograms per gram of raw lyophilized/milled whole seed (dry weight).

Table S2. Iron Concentrations of Pre-Soaked and Cooked Genotypes in the Yellow Bean Panel Organized by Cooking Class.[1]

**Genotype (*Seed Type*)**	**Cooking Class**	**Iron (µg/g)[2]** 2015	2016
Blanco (*white*)	fast	57^{fg}	75^{bcd}
PI527521 (*white*)	fast	71^{c}	79^{b}
Ervilha (*Manteca*)	fast	58^{fg}	73^{cdef}
Cebo (*Manteca*)	fast	61^{ef}	72^{defg}
Mantega (*Manteca*)	fast	61^{fg}	69^{efg}
Uyole 04 (*lt. Amarillo*)	moderate	57^{fg}	67^{ghi}
Chumbo (*Njano*)	moderate	65^{de}	74^{bcd}
Uyole 98 (*dk. Amarillo*)	moderate	60^{fg}	68^{gh}
JB178 (*Red Mottled*)	moderate	85^{ab}	90^{a}
ACC Y012 (*Mayocoba*)	moderate	60^{fg}	71^{defg}
Canario, Cela (*Canary*)	moderate	70^{cd}	74^{cde}
CDC-Sol (*Mayocoba*)	moderate	57^{fg}	63^{i}
DBY28-1 (*Mayocoba*)	moderate	51^{h}	64^{hi}
Y11405 (*Mayocoba*)	moderate	80^{b}	78^{bc}
Canario (*Canary*)	slow	70^{cd}	77^{bc}
PI527538 (*Njano*)	slow	55^{gh}	68^{gh}
PR0737-1 (*Red Mottled*)	slow	89^{a}	86^{a}
Amarelo (*dk. Amarillo*)	slow	65^{de}	76^{bcd}

[1]Values are means of duplicate measurements from two field replicates per genotype (n= 4), measured for field seasons 2015 and 2016. Means sharing the same letter in each column are not significantly different at $P \leq 0.05$. Genotypes are categorized top to bottom from the fastest to slowest cooking entry. [2]Total iron concentration measured as micrograms per gram of cooked lyophilized/milled whole seed (dry weight).

Table S3. Iron Concentrations of Unsoaked and Cooked Genotypes in the Yellow Bean Panel Organized by Cooking Class.[1]

Genotype (*Seed Type*)	Cooking Class	Iron (µg/g)[2] 2015	Iron (µg/g)[2] 2016
Blanco (*white*)	fast	60[efg]	70[bc]
PI527521 (*white*)	fast	69[bc]	70[bc]
Ervilha (*Manteca*)	fast	59[fg]	67[cdef]
Cebo (*Manteca*)	fast	56[ghi]	65[defg]
Mantega (*Manteca*)	fast	59[fgh]	65[defg]
Uyole 04 (*lt. Amarillo*)	moderate	54[hi]	68[bcde]
Chumbo (*Njano*)	moderate	65[cde]	64[efg]
Uyole 98 (*dk. Amarillo*)	moderate	63[def]	66[cdef]
JB178 (*Red Mottled*)	moderate	82[a]	82[a]
Canario, Cela (*Canary*)	moderate	73[b]	67[cde]
Y11405 (*Mayocoba*)	moderate	81[a]	79[a]
DBY28-1 (*Mayocoba*)	moderate	53[i]	62[fgh]
PI527538 (*Njano*)	moderate	56[ghi]	64[efg]
Canario (*Canary*)	slow	68[bc]	73[b]
ACC Y012 (*Mayocoba*)	slow	62[def]	61[gh]
CDC-Sol (*Mayocoba*)	slow	59[fgh]	59[h]
PR0737-1 (*Red Mottled*)	slow	82[a]	81[a]
Amarelo (*dk. Amarillo*)	slow	66[cd]	70[bcd]

[1]Values are means of duplicate measurements from two field replicates per genotype (n = 4), measured for field seasons 2015 and 2016. Means sharing the same letter in each column are not significantly different at $P \leq 0.05$. Genotypes are categorized top to bottom from the fastest to slowest cooking entry. [2]Total iron concentration measured as micrograms per gram of cooked lyophilized/milled whole seed (dry weight).

Table S4. Iron Contents and Retention Values of Pre-Soaked and Cooked Genotypes in the Yellow Bean Panel Organized by Cooking Class.[1]

		**Iron (mg/100 seed)**[2]					
		**2015**			**2016**		
**Genotype (*Seed Type*)**	**Cooking Class**	Raw	Cooked	Retention (%)[3]	Raw	Cooked	Retention (%)
Blanco (*white*)	fast	2.97^{c}	2.55^{c}	86^{abc}	3.88^{a}	3.27^{a}	84^{cdef}
PI527521 (*white*)	fast	2.61^{de}	2.20^{defg}	84^{bcd}	3.19^{b}	2.71^{bcd}	85^{cdef}
Ervilha (*Manteca*)	fast	2.69^{d}	2.34^{cde}	87^{abc}	3.36^{b}	2.92^{b}	87^{abc}
Cebo (*Manteca*)	fast	2.13^{h}	1.82^{h}	85^{abc}	2.23^{g}	1.98^{ij}	89^{abc}
Mantega (*Manteca*)	fast	2.58^{de}	2.15^{efg}	83^{bcde}	2.67^{def}	2.43^{efg}	91^{a}
Uyole 04 (*lt. Amarillo*)	moderate	2.29^{fgh}	2.00^{fgh}	87^{ab}	2.76^{cde}	2.39^{efg}	86^{abcd}
Chumbo (*Njano*)	moderate	2.37^{fg}	1.98^{fgh}	84^{bcd}	2.88^{cd}	2.52^{cdef}	87^{abc}
Uyole 98 (*dk. Amarillo*)	moderate	2.47^{ef}	2.01^{fgh}	81^{def}	2.76^{cde}	2.28^{fgh}	83^{defg}
JB178 (*Red Mottled*)	moderate	3.77^{b}	3.31^{a}	88^{a}	3.67^{a}	3.31^{a}	90^{ab}
ACC Y012 (*Mayocoba*)	moderate	2.74^{d}	2.24^{def}	82^{def}	2.92^{c}	2.49^{def}	85^{bcde}
Canario, Cela (*Canary*)	moderate	3.08^{c}	2.51^{c}	82^{cdef}	2.90^{cd}	2.44^{efg}	84^{cdef}
CDC-Sol (*Mayocoba*)	moderate	2.97^{c}	2.44^{cd}	82^{cdef}	2.78^{cde}	2.29^{fgh}	82^{defg}
DBY28-1 (*Mayocoba*)	moderate	2.20^{gh}	1.78^{h}	81^{defg}	2.47^{fg}	2.01^{ij}	81^{efg}
Y11405 (*Mayocoba*)	moderate	4.12^{a}	3.31^{a}	80^{efg}	3.25^{b}	2.64^{cde}	81^{efg}
Canario (*Canary*)	slow	2.69^{d}	2.13^{efg}	79^{fg}	2.56^{ef}	2.08^{hi}	81^{efg}
PI527538 (*Njano*)	slow	2.48^{ef}	1.97^{gh}	79^{fg}	2.76^{cde}	2.22^{ghi}	80^{fg}
PR0737-1 (*Red Mottled*)	slow	3.65^{b}	3.03^{b}	83^{cde}	3.26^{b}	2.76^{bc}	85^{cdef}
Amarelo (*dk. Amarillo*)	slow	1.84^{i}	1.41^{i}	77^{g}	2.23^{g}	1.75^{j}	79^{g}

[1]Values are means of duplicate measurements from two field replicates per genotype (n = 4), measured for field seasons 2015 and 2016. Means sharing the same letter in each column are not significantly different at $P \leq 0.05$. Genotypes are categorized top to bottom from the fastest to slowest cooking entry. [2]Iron seed content calculated as the total milligrams in 100 intact lyophilized raw or cooked whole seed (dry weight). [3]Retention values calculated by comparing content differences between 100 lyophilized raw and cooked whole seed.

Table S5. Iron Contents and Retention Values of Unsoaked and Cooked Genotypes in the Yellow Bean Panel Organized by Cooking Class.[1]

		**Iron (mg/100 seed)[2]**					
		**2015**			**2016**		
**Genotype (*Seed Type*)**	**Cooking Class**	Raw	Cooked	Retention (%)[3]	Raw	Cooked	Retention (%)
Blanco (*white*)	fast	3.33^{bc}	2.72^{bc}	82^{abc}	3.64^{a}	2.91^{a}	80^{abc}
PI527521 (*white*)	fast	2.87^{fgh}	2.31^{def}	81^{abc}	3.02^{cd}	2.55^{bc}	80^{abc}
Ervilha (*Manteca*)	fast	3.06^{def}	2.59^{bc}	84^{ab}	3.19^{bc}	2.64^{bc}	83^{ab}
Cebo (*Manteca*)	fast	2.27^{k}	1.82^{h}	80^{abc}	2.65^{fghi}	2.13^{ef}	80^{abc}
Mantega (*Manteca*)	fast	2.60^{ij}	2.14^{fg}	82^{abc}	2.59^{hi}	2.19^{de}	84^{a}
Uyole 04 (*lt. Amarillo*)	moderate	2.45^{jk}	1.99^{gh}	81^{abc}	2.63^{ghi}	2.21^{de}	84^{a}
Chumbo (*Njano*)	moderate	2.45^{jk}	2.00^{gh}	82^{abc}	2.43^{i}	1.97^{f}	81^{abc}
Uyole 98 (*dk. Amarillo*)	moderate	2.70^{hi}	2.16^{fg}	80^{bcd}	2.85^{defg}	2.25^{de}	79^{bcd}
JB178 (*Red Mottled*)	moderate	3.25^{bcd}	2.77^{b}	85^{a}	3.33^{b}	2.73^{ab}	82^{abc}
Canario, Cela (*Canary*)	moderate	3.42^{b}	2.69^{bc}	79^{cd}	2.88^{def}	2.22^{de}	77^{def}
Y11405 (*Mayocoba*)	moderate	4.12^{a}	3.18^{a}	77^{cd}	3.40^{ab}	2.67^{ab}	78^{cde}
DBY28-1 (*Mayocoba*)	moderate	2.26^{k}	1.76^{h}	78^{cd}	2.64^{fghi}	2.04^{ef}	77^{def}
PI527538 (*Njano*)	moderate	2.85^{gh}	2.20^{fg}	77^{cd}	2.78^{efgh}	2.06^{ef}	74^{ef}
Canario (*Canary*)	slow	2.94^{efg}	2.22^{efg}	75^{de}	2.94^{de}	2.15^{de}	73^{ef}
ACC Y012 (*Mayocoba*)	slow	2.79^{ghi}	2.19^{fg}	78^{cd}	2.62^{ghi}	2.09^{ef}	80^{abc}
CDC-Sol (*Mayocoba*)	slow	3.11^{de}	2.47^{cde}	79^{bcd}	2.73^{efgh}	2.13^{ef}	79^{bcd}
PR0737-1 (*Red Mottled*)	slow	3.21^{cd}	2.50^{cd}	76^{cd}	3.29^{b}	2.55^{bc}	77^{cde}
Amarelo (*dk. Amarillo*)	slow	1.80^{l}	1.29^{i}	71^{e}	2.16^{j}	1.56^{g}	72^{f}

[1]Values are means of duplicate measurements from two field replicates per genotype (n = 4), measured for field seasons 2015 and 2016. Means sharing the same letter in each column are not significantly different at $P \leq 0.05$. Genotypes are categorized top to bottom from the fastest to slowest cooking entry. [2]Iron seed content calculated as the total milligrams in 100 intact lyophilized raw or cooked whole seed (dry weight). [3]Retention values calculated by comparing content differences between 100 lyophilized raw and cooked whole seed.

Table S6. Iron Bioavailability Scores of Pre-Soaked and Cooked Genotypes in the Yellow Bean Panel Organized by Cooking Class.[1]

		Iron Bioavailability (% control)[2]	
**Genotype (*Seed Type*)**	**Cooking Class**	2015	2016
Blanco (*white*)	fast	101[ab]	97[bc]
PI527521 (*white*)	fast	107[a]	100[bc]
Ervilha (*Manteca*)	fast	100[ab]	105[b]
Cebo (*Manteca*)	fast	101[ab]	136[a]
Mantega (*Manteca*)	fast	107[a]	104[bc]
Uyole 04 (*lt. Amarillo*)	moderate	74[cd]	87[bcd]
Chumbo (*Njano*)	moderate	63[de]	58[efg]
Uyole 98 (*dk. Amarillo*)	moderate	61[def]	50[fg]
JB178 (*Red Mottled*)	moderate	43[fg]	45[fgh]
ACC Y012 (*Mayocoba*)	moderate	66[de]	60[ef]
Canario, Cela (*Canary*)	moderate	60[def]	43[fgh]
CDC-Sol (*Mayocoba*)	moderate	57[def]	47[fgh]
DBY28-1 (*Mayocoba*)	moderate	67[cde]	71[de]
Y11405 (*Mayocoba*)	moderate	84[bc]	86[cd]
Canario (*Canary*)	slow	55[ef]	41[gh]
PI527538 (*Njano*)	slow	34[gh]	44[fgh]
PR0737-1 (*Red Mottled*)	slow	34[gh]	29[hi]
Amarelo (*dk. Amarillo*)	slow	19[h]	22[i]

[1]Values are means of triplicate measurements from two field replicates per genotype (n = 6), measured for field seasons 2015 and 2016. Means sharing the same letter in each column are not significantly different at $P \leq 0.05$. Genotypes are categorized top to bottom from the fastest to slowest cooking entry. [2]*In vitro* iron bioavailability is expressed as a percentage score of Caco-2 cell ferritin formation (ng ferritin / mg total cell protein) that is relative to a white navy bean (cv. Merlin) control, following *in vitro* digestion of lyophilized cooked whole seed.

Table S7. Iron Bioavailability Scores of Unsoaked and Cooked Genotypes in the Yellow Bean Panel Organized by Cooking Class.[1]

		Iron Bioavailability (% control)[2]	
**Genotype (*Seed Type*)**	**Cooking Class**	2015	2016
Blanco (*white*)	fast	103[cd]	97[bcd]
PI527521 (*white*)	fast	125[b]	104[b]
Ervilha (*Manteca*)	fast	124[b]	125[a]
Cebo (*Manteca*)	fast	109[bc]	111[ab]
Mantega (*Manteca*)	fast	159[a]	100[bc]
Uyole 04 (*lt. Amarillo*)	moderate	86[de]	79[def]
Chumbo (*Njano*)	moderate	64[fg]	69[efg]
Uyole 98 (*dk. Amarillo*)	moderate	63[fg]	50[hi]
JB178 (*Red Mottled*)	moderate	33[h]	50[hi]
Canario, Cela (*Canary*)	moderate	66[fg]	50[hi]
Y11405 (*Mayocoba*)	moderate	80[ef]	76[ef]
DBY28-1 (*Mayocoba*)	moderate	87[de]	84[cde]
PI527538 (*Njano*)	moderate	34[h]	62[fgh]
Canario (*Canary*)	slow	55[g]	51[ghi]
ACC Y012 (*Mayocoba*)	slow	79[ef]	47[hi]
CDC-Sol (*Mayocoba*)	slow	69[f]	57[ghi]
PR0737-1 (*Red Mottled*)	slow	24[h]	41[ij]
Amarelo (*dk. Amarillo*)	slow	20[h]	24[j]

[1]Values are means of triplicate measurement from two field replicates per genotype (n = 6), measured for field seasons 2015 and 2016. Means sharing the same letter in each column are not significantly different at $P \leq 0.05$. Genotypes are categorized top to bottom from the fastest to slowest cooking entry. [2]*In vitro* iron bioavailability is expressed as a percentage score of Caco-2 cell ferritin formation (ng ferritin / mg total cell protein) that is relative to a white navy bean (cv. Merlin) control, following *in vitro* digestion of lyophilized cooked whole seed.
